# Timosaponin AIII Induces G2/M Arrest and Apoptosis in Breast Cancer by Activating the ATM/Chk2 and p38 MAPK Signaling Pathways

**DOI:** 10.3389/fphar.2020.601468

**Published:** 2021-01-15

**Authors:** Minjie Zhang, Jiaxi Qu, Zhiwei Gao, Qi Qi, Hong Yin, Ling Zhu, Yichen Wu, Wei Liu, Jian Yang, Xuefeng Huang

**Affiliations:** ^1^ Department of Natural Medicinal Chemistry, School of Chinese Pharmacy, China Pharmaceutical University, Nanjing, China; ^2^ Department of Pharmacology, Nanjing Medical University, Nanjing, China; ^3^ MOE Key Laboratory of Tumor Molecular Biology, Clinical Translational Center for Targeted Drug, Department of Pharmacology, School of Medicine, Jinan University, Guangzhou, China

**Keywords:** timosaponin AIII, breast cancer, G2/M arrest, DNA damage response, p38 MAPK signaling

## Abstract

Timosaponin AIII (TAIII), a steroidal saponin, exerts potent anti-tumor activity in various cancers, especially breast cancer. However, the concrete molecular mechanisms of TAIII against breast cancer are still unclear. Here, we find that TAIII triggers DNA damage, leads to G2/M arrest, and ultimately induces apoptosis in breast cancer both *in vitro* and *in vivo*. TAIII induced G2/M phase arrest and apoptosis in MDA-MB-231 and MCF7 cells accompanied with down-regulation of CyclinB1, Cdc2 and Cdc25C. Further data showed that the ATM/Chk2 and p38 pathways were activated representing by up-regulated levels of p-H2A.X and p-p38, which indicated an induction of DNA damage by TAIII, leading to cell cycle arrest and apoptosis. The effects of TAIII were further confirmed by employing inhibitors of ATM and p38 pathways. *In vivo*, TAIII suppressed the growth of subcutaneous xenograft tumor without obvious toxicity, which indicated by Ki67 and TUNEL analysis. Data also showed that TAIII stimulated the ATM/Chk2 and p38 MAPK pathways *in vivo*, which in consistent with the effects *in vitro*. Hence, our data demonstrate that TAIII triggers DNA damage and activates ATM/Chk2 and p38 MAPK pathways, and then induces G2/M phase arrest and apoptosis in breast cancer, which provide theoretical evidence for TAIII utilized as drug against breast cancer.

## Introduction

Breast cancer is one of the most common neoplasms worldwide, which occurs mostly in women aged 15–59 years (He and Chen, 2012; Chen et al., 2018). Many breast cancer patients presented distinct metastasis, including lymph nodes, lung liver, and bone marrow (Muller et al., 2001). Systemic treatment of breast cancer includes cytotoxic, hormonal, and immunotherapeutic agents (Gonzalez-Angulo et al., 2007). However, there is little evidence-based consensus on when to use which treatments, in what combination and for how long (Stockler et al., 2000). Despite exciting progress in the understanding of breast cancer development and progression, and in the development of novel therapeutic strategies in recent years, according to statistics, breast cancer is still the most common cancer diagnosed among women and is the second leading cause of cancer death among women after lung cancer in United States by 2020 (Siegel et al., 2020). Therefore, breast cancer remains a considerable health burden and development of novel therapy against breast cancer is urgent.

The growth and reproduction of eukaryotes rely on the correct replication and distribution of genetic information within the cell cycle progression. Cell cycle is a ubiquitous and complex process involving various physiology and pathology events including cancer development. Tumorigenesis is a multi-step process accompanied by cell cycle disorders (Collins and Garrett, 2005). It is usually observed that the signal that the cell decides to divide is always “open”, whereas the relevant pathways that inhibit cell cycle are “closed”, thereby leading to the cell cycle dysregulated, producing uncontrolled cell proliferation and apoptosis (Schafer, 1998). Proteins involved in the mitotic signal transduction pathway are recognized as oncoproteins including extracellular secreted growth factors, growth factor receptors, cytoplasmic signal transduction cascades and transcription factors (Schafer, 1998). Given the close relationship between the cell cycle and cancer development, targeting the cell cycle has become one of the key strategies for cancer treatment.

*Anemarrhena asphodeloide* (AA), a traditional Chinese medicine, has the effects on clearing heat and purging fire, nourishing yin and moisturizing, quenching thirst and removing annoyance. Timosaponin AIII (TAIII), a steroidal saponin, is a major active component of AA (King et al., 2009), which exerts various pharmacological activities including antipyretic, anti-inflammatory (Lim et al., 2015), antidiabetic (Kimura et al., 1996), antidepressive (Ren et al., 2006), and improvement of learning and memory (Hu et al., 2005). Recently, TAIII has showed potent anti-tumor activity against various cancers *in vivo* and *in vitro*, such as gastric cancer, cervical cancer, colorectal cancer, breast cancer, acute leukemia, non-small cell lung cancer, melanoma, liver cancer, and pancreatic cancer, etc (Takeda et al., 2001; Sy et al., 2008; Tsai et al., 2013; Huang et al., 2015; Jung et al., 2016; Wang et al., 2017). It has been reported that TAIII induces cell cycle arrest in different cancer cells, such as colorectal cancer cells, non-small-cell lung cancer cells and pancreatic cancer cells (Kang et al., 2011; Chen et al., 2016; Jung et al., 2016). However, the precise molecular mechanism is still poorly understood. Besides, TAIII has a potent cytotoxicity in breast cancer and exerts less inhibition in non-transformed cells (King et al., 2009), which makes it an attractive candidate for development as a cancer therapeutic agent against breast cancer. Here, we attempted to examine the effects of TAIII on the cell cycle in breast cancer cell lines and study the underlying mechanisms of action *in vitro* and *in vivo*.

## Materials and Methods

### Materials

Timosaponin AIII (TAIII) was extracted from dry roots of *Anemarrhena asphodeloide*. purchased from Anhui Bozhou Yihongtang Pharmaceutical Co., Ltd (Bozhou, Anhui, China), and identified by Professor Minjian Qin, China Pharmaceutical University, in March 2014. A voucher specimen (No. ZM 20141020) was deposited in Department of Natural Medicinal Chemistry, China Pharmaceutical University. TAIII was isolated from *A. asphodeloides* according to the protocols reported previously (Wang et al., 2017; Zhang et al., 2017). TAIII (purity >98%) was dissolved in DMSO to 10 mM as a stock solution and stored at −20°C. Ki67, β-actin, Histone H2A.X (S130), ATM, Chk2, Bax, GAPDH-antibodies were purchased from Bioworld (Bloomington, MN, United States). Antibody against Histone H3 was purchased from Signalway Antibody (College Park, MD, United States). Antibodies against CyclinB1, Cdc25C, Cdc2, p38, *p*-ser1981-ATM, *p*-thr68-Chk2, *p*-ser216-Cdc25C, *p*-tyr15-Cdc2, *p*-ser10-Histone H3, *p*-ser139-Histone H2A.X, *p*-thr180/tyr182-p38, p21, caspase3, and Bcl-2 were purchased from Cell signaling technology (Danvers, MA, United States). KU55933 (the inhibitor of *p*-ATM), SB203580 (inhibitor of p-p38) were purchased from Selleck (Houston, TX, United States). Doxorubicin hydrochloride was purchased from Sigma-Aldrich.

### Cell Lines and Cell Culture

Human breast cancer cells (MDA-MB-231, MCF-7) were purchased from the Chinese Academy of Sciences (Shanghai, China). Cells were cultured in Dulbecco’s modified Eagle's medium (DMEM) (Gibco, Grand Island, United States) containing 10% fetal bovine serum (FBS) (Hyclone, United States) and 25 μM HEPES. Human mammary epithelial cell line MCF10A was purchased from Nanjing saihongrui Biotechnology Co., Ltd. (Nanjing, China) and cultured in DMEM/Ham’s F-12 supplemented with 100 ng/ml cholera toxin, 20 ng/ml epidermal growth factor (EGF), 0.01 mg/ml insulin, 500 ng/ml hydrocortisone, and 5% chelex-treated horse serum. Cells were incubated at 37°C in a humidified atmosphere with 5% CO_2_. The cells in logarithmic growth phase were collected for further experiments, when MDA-MB-231, MCF-7, and MCF10A cells covered 80–90% bottom of the culture dish.

### Analysis of Cell Cycle

The distribution of cell cycle was analyzed with PI staining by flow cytometry. MDA-MB-231 and MCF-7 cells were treated with/without TAIII (10 μM, 15 μM), TAIII (15 μM) + KU55933 (the inhibitor of *p*-ATM, cells were pretreated with 10 μM for 2 h), TAIII (15 μM) +SB203580 (the inhibitor of p-p38, cells were pretreated with 10 μM for 2 h). Following the treatment, cells were fixed by cold 75% ethanol at −20°C for overnight. The cells were then stained with PI/RNase buffer (BD Bioscience, San Diego, CA, United States). The cell cycle distribution was measured by FACScan laser flow cytometer (FACSCalibur, Becton Dickinson) or MACSQuant Analyzer 10 (Miltenyi Biotec, Bergisch Gladbach Germany), data analyzed with software MODFIT and CELLQUEST (Becton Dickinson, Franklin Lakes, NJ) or FlowJo_V10 (BD Bioscience, San Diego, CA, United States) software.

### Analysis of Cell Apoptosis

Cell apoptosis induced by TAIII was detected by Annexin-V-FITC/PI apoptosis detection kit (BD Bioscience, San Diego, CA, United States). Following treatment with/without different concentrations of TAIII for 24 h, the cells were harvested with (EDTA)-free trypsin and rinsed with PBS twice followed by a centrifugation at 1000 rpm for 5 min at 4°C. The cells were resuspended by binding buffer and then stained with Annexin V-FITC and PI successively in dark for 15 min at room temperature. The apoptosis was measured by MACSQuant Analyzer 10 (Miltenyi Biotec, Bergisch Gladbach Germany) and data were analyzed with FlowJo_V10 (BD Bioscience, San Diego, CA, United States) software.

### Immunofluorescence Analysis

Cells were treated with/without TAIII (15 μM) and doxorubicin (10 μM) for 24 h respectively. Then, the cells were fixed with 4% paraformaldehyde for 10 min and permeabilized with 0.2% Triton X-100 for 10 min at room temperature. After blocked with 5% bovine serum albumin (BSA) for 30 min, the cells were incubated with p-Histone H2AX (ser139) antibody (1:400, dilute in PBST) at 4°C for overnight. And then cells were processed with Tetramethylrhodamine (TMR) labeled anti-rabbit IgG antibody (1:200, dilute in PBST) at 37°C for 1 h followed by incubation with DAPI for 20 min in the dark. Finally the cells were examined by fluorescent microscope (Olympus, Japan) (Acquisition software: DP2-BSW). For the immunofluorescence quantification, the ImageJ software was used for counting the red positive cells, which were normalized to the nuclei (blue).

### Western Blotting Analysis

Cells were lyzed by RIPA buffer at 4°C after rinsed with PBS twice. Tissue amples were minced and mixed well by homogenizer with RIPA buffter for protein textraction followed by quantification with BCA method (Bioworld, Bloomington, MN, United States). Total proteins were separated by sodium dodecyl sulfate-polyacrylamide gel electrophoresis (SDS-PAGE) (Bio-Rad, Hercules, CA, United States) and transferred to PVDF membrane (Bio-Rad, Hercules, CA, United States). The membranes were incubated with primary antibody for overnight at 4 °C after blocking with 5% skim milk (formulated by TBST) at room temperature for 1–1.5 h. The membranes were then incubated with corresponding second-antibody for 1 h after rinsed with TBST three times, 10 min for each time. Following incubation with the ELC reagent (a mixture of A and B solution in equal proportion) (Vazyme biotech, Nanjing, China). the membranes were visualized by imaging system (CLiNX Scientific Instrument Co., Ltd., Shanghai, China). Blots were analyzed with ImageJ software.

### Xenograft Tumors in Nude Mice

Female BALB/c-Nude mice (5–6 weeks, 19–22 g, SPF grade) were purchased from Cavens Experimental Animal Technology Co., Ltd. (Changzhou, China). The mice were raised in air conditioned rooms under controlled lighting (12 h lighting/d) and were fed with standard laboratory food and water ad libitum. The ethical committee approval No. is IACUC1911001. 150 μL media containing 5×10^6^ MDA-MB-231 cells were injected subcutaneously into the right flanks of female BALB/c-Nude mice to establish subcutaneous xenograft models. The mice were grouped randomly into three groups, six mice per group, when the tumor volume was about 100 mm^3^ (the tumor size was measured with by a digital caliper and the formula for calculating the size of the tumor is V = length×width^2^/2). Then the three groups of mice were treated with vehicle (v/v) (1% tween 80, 2% DMSO, 97% physiological saline), 5 mg/kg TAIII and 10 mg/kg TAIII every other day by intraperitoneal injection, respectively. Weights of the mice and the volume of tumor were recorded every other day. The mice were sacrificed and tumor tissues were collected when the volume of the tumors in the vehicle group reached 1000 mm^3^. The tumor inhibition rate was calculated as (W_V_–W_T_)/W_V_×100%, where W_V_ and W_T_ are the mean weights of tumors in the vehicle group and drug treated groups.

### Immunohistochemistry

Tumor sections of paraffin-embedded were dewaxed to water and repaired with repair solution and rinsed with PBS three times, 5 min for each time. Sections were incubated in 3% H_2_O_2_ at room temperature for 15 min and rinsed with PBS three times, 5 min for each time. Then the sections were blocked with 10% normal goat serum (diluted in PBS) followed by incubation with primary antibody (Ki67, 1:200, dilute in goat serum) for overnight at 4 °C. Sections were added second antibody and incubate at 37°C for 1 h after rinsed with PBS three times, 5 min for each time. Keeping the tissue moist, add 100 μl of DAB staining solution to each sample in the dark. Images were taken by fluorescence microscope (Olympus, Japan) (Acquisition software: DP2-BSW). For the immunohistochemistry quantification, the ImageJ software was used for counting the positive cells.

### Hematoxylin and Eosin Staining

The histological alterations of organ tissues were observed by H&E staining. Heart, liver, spleen, lung, and kidney were fixed in 4% paraformaldehyde for overnight and embedded in paraffin. The organ tissues embedded in paraffin were sliced into 5 μm and hydrated with gradient concentration of ethanol. The tissues were stained with hematoxylin and eosin (H&E) to evaluate the histological alterations by digital microscope (Olympus, Japan) (Acquisition software: DP2-BSW).

### TUNEL Assay

TdT-UTP nick end labeling (TUNEL) assay was performed using the one-step TUNEL apoptosis assay kit (KeyGEN, Nangjing, China). According to the manufacturer’s instructions, after deparaffinization and rehydration, the paraffin-embedded tumor sections were incubated with proteinase K at room temperature for 20 min followed by rinsed with PBS. Then the sections were processed with TUNEL reaction buffer at 37°C for 1 h, followed by incubated with DAPI for 10 min in the dark. Finally the sections were examined by fluorescent microscope (Olympus, Japan) (Acquisition software: DP2-BSW). For the immunofluorescence quantification, the ImageJ software was used for counting the red positive cells and normalizing to the nuclei number (blue).

### Statistical Analysis

SPSS 19.0 software (IBM, United States) was employed for statistical analysis. The experimental data were expressed in mean ± standard deviation and the differences between groups was analyzed by *t*-test, two-way analysis of variance (ANOVA) and Turkey multiple comparison. *p* < 0.05 was considered statistically significant, and *p* values were labeled as follows: *p < 0.05; **p < 0.01; ***p < 0.001. Each experiment was repeated at least three times.

## Results

### TAIII Induces G2/M Arrest in Breast Cancer Cells

**FIGURE 1:**
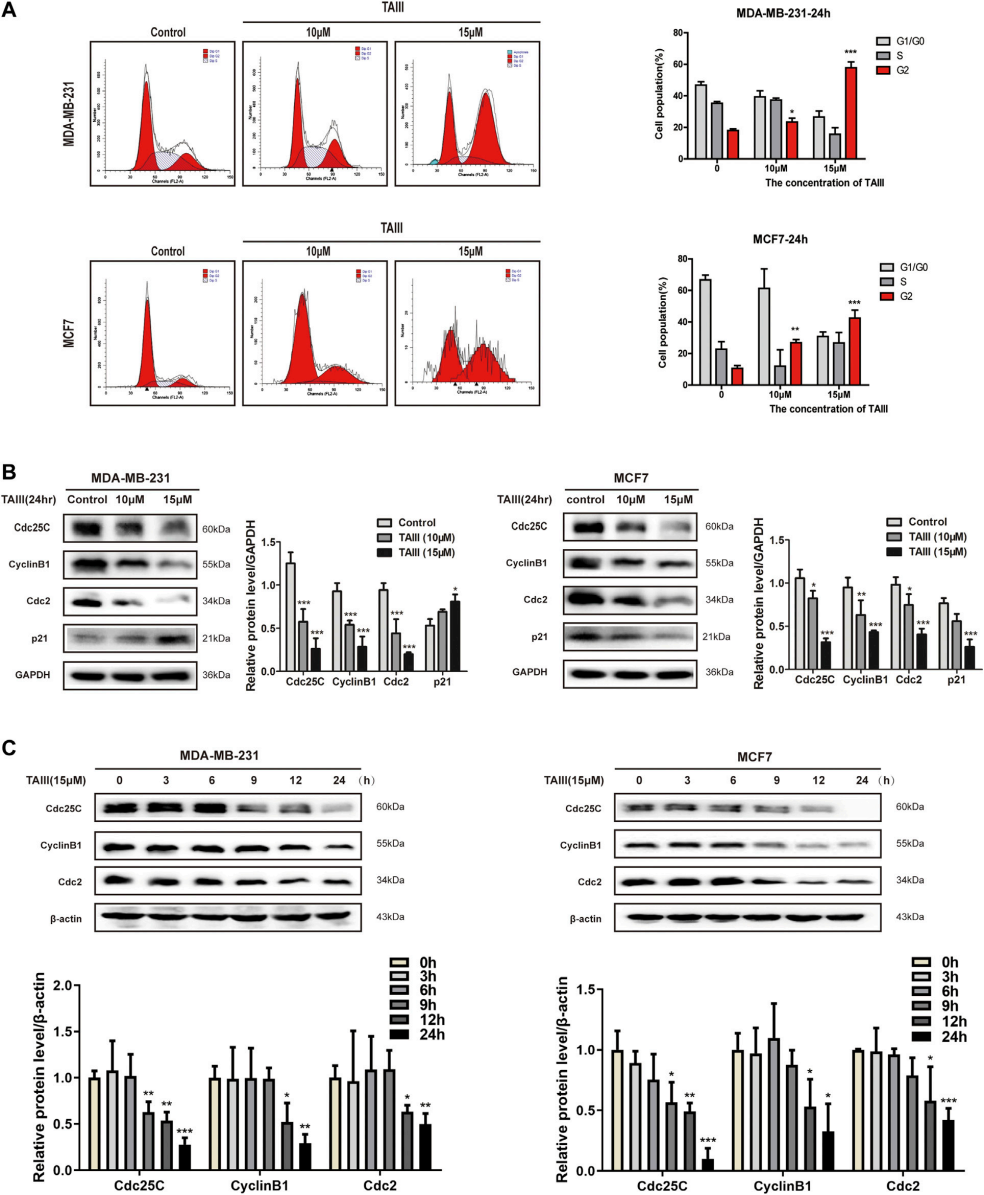
TAIII induces G2/M arrest in breast cancer cells. **(A)** MDA-MB-231 and MCF-7 cells were treated with indicated concentration of TAIII or medium for 24 h respectively and cell cycle distribution (percent cells) was analyzed by FACScan laser flow cytometer after PI-staining, and data analyzed with software MODFIT and CELLQUEST. **(B)** MDA-MB-231 and MCF-7 cells were treated with indicated concentration of TAIII for 24 h. The total cell lysates were prepared and the levels of Cdc25c, CyclinB1, Cdc2, and p21 were detected by western blot analysis. **(C)** MDA-MB-231 and MCF-7 cells were treated with 15 μM TAIII for indicated time and the expression of same proteins were assayed by western blot analysis. GAPDH and β-actin were used as the loading control respectively. The data were expressed as the mean ± SD of the results from three separate experiments and the differences between groups was analyzed by *t*-test, two-way analysis of variance (ANOVA). *p < 0.05; **p < 0.01; ***p < 0.001.

In order to clarify the exact mechanism of TAIII against breast cancer, effect of TAIII on the cell cycle was detected. Human breast cancer MDA-MB-231 and MCF-7 cells were treated with 10 and 15 μM TAIII for 24 h respectively and stained with PI after fixed with 75% ethanol. Then the distributions of cell cycle were analyzed by flow cytometry (FCM). Results showed that the percentage of MDA-MB-231 cells in G2/M phase was increased from 17.99% (Control) to 23.35% (TAIII 10 μM) and 57.8% (TAIII 15 μM) (Figure 1A). Meanwhile, the percentages of MCF-7 cells in G2/M phase were 10.65% (Control), 26.90% (TAIII 10 μM), and 42.49% (TAIII 15 μM) respectively (Figure 1A). These results demonstrat that TAIII induces cell cycle arrest at G2/M phase in breast cancer cells in a concentration-dependent manner.

Cell cycle regulated by various regulatory proteins including cyclins and their kinase partners-cyclin dependent kinase (CDKs). Among them, Cdc25C, CyclinB1 and Cdc2 are crucial regulators at G2/M phase. Following treatment with TAIII, expressions of Cdc25C, CyclinB1, and Cdc2 were all decreased in concentration- and time-dependent manners (Figures 1B,C). However, changes of p21 expression were different in the two cell lines. It is noticed that TAIII-mediated protein changes were observed as early as 9 h after treatment, and persisted for the duration of the experiment. These data indicate that TAIII induces G2/M phase arrest of breast cancer cells accompanied by down-regulating cycle-related proteins Cdc25C, CyclinB1, and Cdc2.

### TAIII Induces Apoptosis in Breast Cancer Cells

**FIGURE 2:**
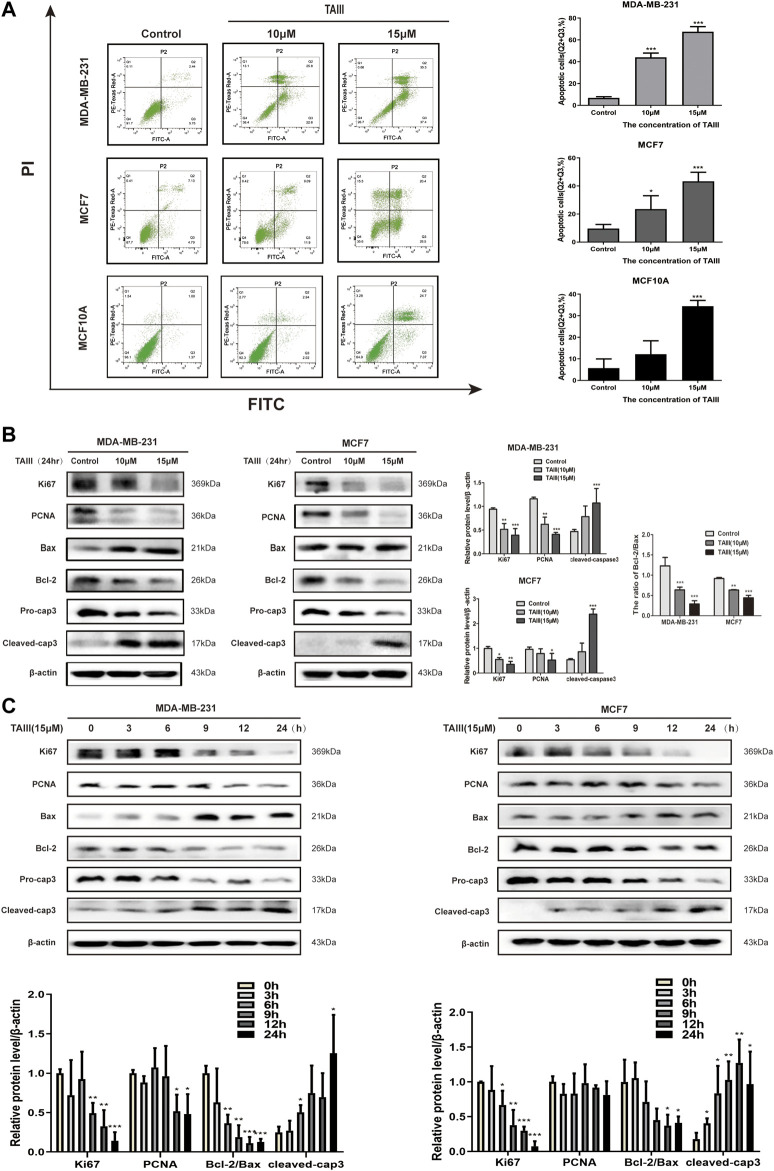
TAIII induces apoptosis in breast cancer cells. **(A)** MDA-MB-231, MCF-7 and MCF10A cells were treated with indicated concentration of TAIII or medium for 24 h respectively and the apoptosis was analyzed by flow cytometry after stained with Annexin-V-FITC/PI. **(B)** MDA-MB-231 and MCF-7 cells were treated with indicated concentration of TAIII for 24 h. The total cell lysates were prepared and the levels of Ki67, PCNA, Bax, Bcl-2 and Caspase-3 were detected by western blot analysis. **(C)** MDA-MB-231 and MCF-7 cells were treated with 15 μM TAIII for indicated time and the expression of same proteins was assayed by western blot analysis. β-actin was used as the loading control. The data were expressed as the mean ± SD of the results from three separate experiments and the differences between groups was analyzed by *t*-test, two-way analysis of variance (ANOVA). *p < 0.05; **p < 0.01; ***p < 0.001.

Since apoptosis is a common outcome of cell cycle arresting, apoptotic cells induced by TAIII were detected by FCM after staining with Annexin-V-FITC/PI. As shown in Figure 2A, both early apoptosis (Annexin V positive, PI negative) and late apoptosis (Annexin V positive, PI positive) of the breast cancer cells were significantly increased in a concentration-dependent manner with the rates increasing from 5.9% (Control) to 44.0% (TAIII 10 μM) and 67.5% (TAIII 15 μM), respectively. For MCF-7 cells, the corresponding apoptosis rates of Control, TAIII (10 μM) and TAIII (15 μM) were 9.5%, 23.5%, and 43.3%, respectively. We also examined the apoptosis induction of TAIII in human mammary epithelial cell line MCF10A. As shown in Figure 2A, the corresponding apoptosis rates of Control, TAIII (10 μM) and TAIII (15 μM) were 5.6%, 12.1%, and 34.3%, respectively.

Further, we examined the expressions of apoptosis- and proliferation-related proteins in MDA-MB-231 and MCF7 cells after treatment with TAIII. The results showed that TAIII down-regualted the levles of proliferation-related proteins (Ki67, PCNA) in a concentration-dependent manner (Figure 2B). Meanwhile, a decreased in the Bcl-2/Bax ratio was also observed in two cell lines, which was accompanied by decreased pro-caspase 3 and increased cleaved-caspase 3. The regulaitons of the proteins described above by TAIII were also in a time-dependent manner (Figure 2C). These results indicate that TAIII inhibits cell proliferation and induces apoptosis of breast cancer cells by regulating the expression of apoptosis- and proliferation-related proteins.

### TAIII Activates the ATM/Chk2/Cdc25C Pathway and ATM Specific Inhibitor Partially Attenuates TAIII-Induced G2 Arrest in Breast Cancer Cells

**FIGURE 3:**
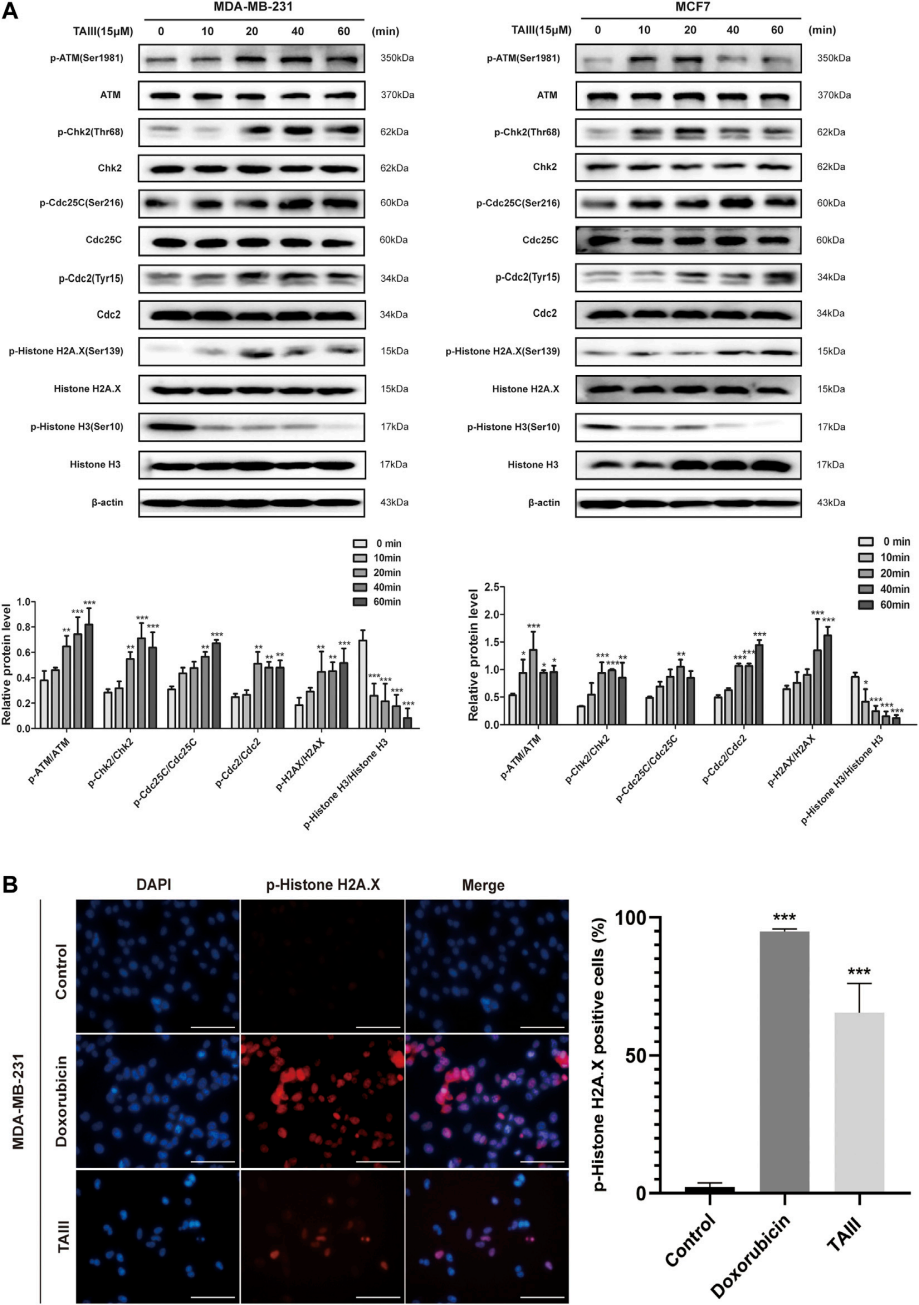
TAIII induces DNA damage and activates the ATM/Chk2/Cdc25C pathway. **(A)** MDA-MB-231 and MCF-7 cells were treated with 15 μM TAIII for 0, 10, 20, 40, and 60 min respectively and the expressions of indicated proteins were detected by western blot. β-actin was used as the loading control. **(B)** MDA-MB-231 cells were treated with 15 μM TAIII and 10 μM doxorubicin for 24 h respectively, and immunofluorescence assay was performed to detect DNA damage marker p-Histone H2A.X. For each panel, the images (left to right) show nuclei stained by DAPI (blue), p-Histone H2A.X (red), and overlays of the two images. Scale bar = 50 μm and magnification = ×400. The data were expressed as the means ± SD of the results from three separate experiments and the differences between groups was analyzed by *t*-test, two-way analysis of variance (ANOVA). *p < 0.05; **p < 0.01; ***p < 0.001.

It has been demonstrated that Cdc2 is essential for the progression from G2 to mitosis phases, and the increase of Cdc2 inhibitory phosphorylation at Tyr15 is a hallmark of G2/M arrest. Fowllowing treatment with 15 μM TAIII, an increased phosphorylation of Cdc2 at Tyr15 was observed in a time-dependent manner both in MDA-MB-231 and MCF7 cells (Figure 3A). Cdc25C is able to dephosphorylate the Cdc2 at Tyr15 and Thr14 and active the kinase activity of Cdc2 (Hoffmann and Karsenti, 1994; Schafer, 1998). Meanwhile, a time-dependent increase of inhibitory phosphorylation of Cdc25C at Ser216 was observed after treatment with TAIII, which failed to restore the activity of Cdc2.

To further investigate which pathways are involved in TAIII-induced G2/M arrest, we analyzed the upstream regulators of Cdc25C. As shown in Figure 3A, ATM is activated by phosphorylation at Ser1981 after TAIII treatment for 10–20 min, accompanied by activation of Chk2 (phosphorylation at Thr68), a main target of ATM. ATM/Chk2 is one of the main activation pathways of DNA damage response (DDR). DNA damage is an important reason for G2/M phase arrest (Maity et al., 1994). The increase of the phosphorylation of H2AX at Ser139 (γ-H2AX), a marker of DNA damage repair, was also observed after TAIII treatment, which indicated that TAIII might induce DNA damage in breast cancer cells.These results suggest that TAIII-induced DNA damage activates the DDR pathway, ATM/Chk2 pathway, and causes cell cycle arrest in G2/M phase. Besides, decreased expression of phosphorylation of Histone H3 at Ser10 which plays an essential role in chromatin remodeling, was observed, indicating that TAIII induced DNA damage through chromatin remodeling. Next, immunofluorescence assay was emploied to detect the change of γ-H2AX after MDA-MB-231 cells were treated with TAIII (15 μM) and doxorubicin (10 μM) for 24 h. The induction of nuclear γ-H2AX signals and formation of γ-H2AX foci were visualized both in doxrubicin (10 μM) and TAIII (15 μM) treatment cells (Figure 3B).

**FIGURE 4:**
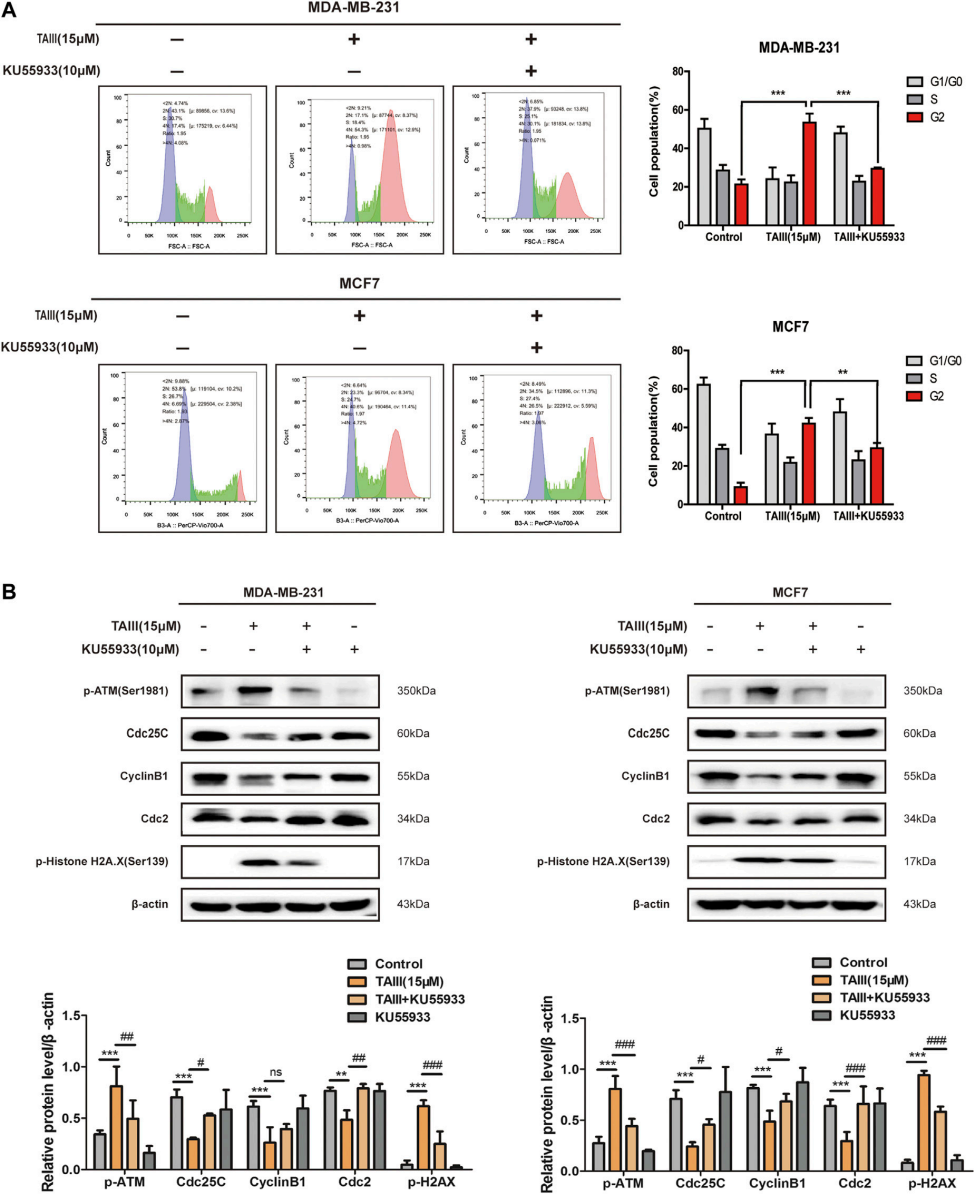
ATM specific inhibitor partially attenuates TAIII-induced G2 arrest in breast cancer cells. **(A)** MDA-MB-231 and MCF-7 cells were pretreated with/without 10 μM KU55933 for 2 h before treated with 15 μM TAIII and the cell cycle distribution (percent cells) was analyzed by MACSQuant Analyzer 10 after PI-staining, and data analyzed with FlowJo_V10 software. **(B)** MDA-MB-231 and MCF-7 cells were pretreated with/without 10 μM KU55933 for 2 h before treated with 15 μM TAIII and the expression of *p*-ATM, p-Histone H2A.X, Cdc25C, CyclinB1 and Cdc2 were detected by western blot. β-actin was used as the loading control. The data were expressed as the means ± SD of the results from three separate experiments and the differences between groups was analyzed by *t*-test, two-way analysis of variance (ANOVA). *p < 0.05; **p < 0.01; ***p < 0.001.

To further confirm the role of the ATM/Chk2 pathway in TAIII-induced G2/M phase arrest, MDA-MB-231 and MCF7 cells were pretreated with/without 10 μM KU55933 (a specific inhibitor of *p*-ATM) for 2 h before TAIII treatment. FCM results showed that TAIII-induced G2/M phase arrest was partially reversed by blocking the ATM/Chk2 pathway (Figure 4A). The percentage of MDA-MB-231 cells in G2/M phase was decreased from 53.43% in the TAIII group to 29.45% in the TAIII+KU55933 group (Figure 4A). For MCF7 cells, the percentage of cells in G2/M phase was decreased from 41.94% in the TAIII group to 29.25% in the TAIII+KU55933 group (Figure 4A). Meanwhile, the alternations of related proteins in the cell cycle were detected by western blot. TAIII-induced changes of Cdc25C, CyclinB1, Cdc2 and γ-H2AX were partially restored by KU55933 pretreatment (Figure 4B). These data demonstrate that activation ATM/Chk2 pathway involves in TAIII-induced G2/M arrest in breast cancer cells.

### TAIII Activates the p38 MAPK Signaling Pathway and p38 Specific Inhibitor Partial Attenuates TAIII-Induced G2 Arrest in Breast Cancer Cells

**FIGURE 5:**
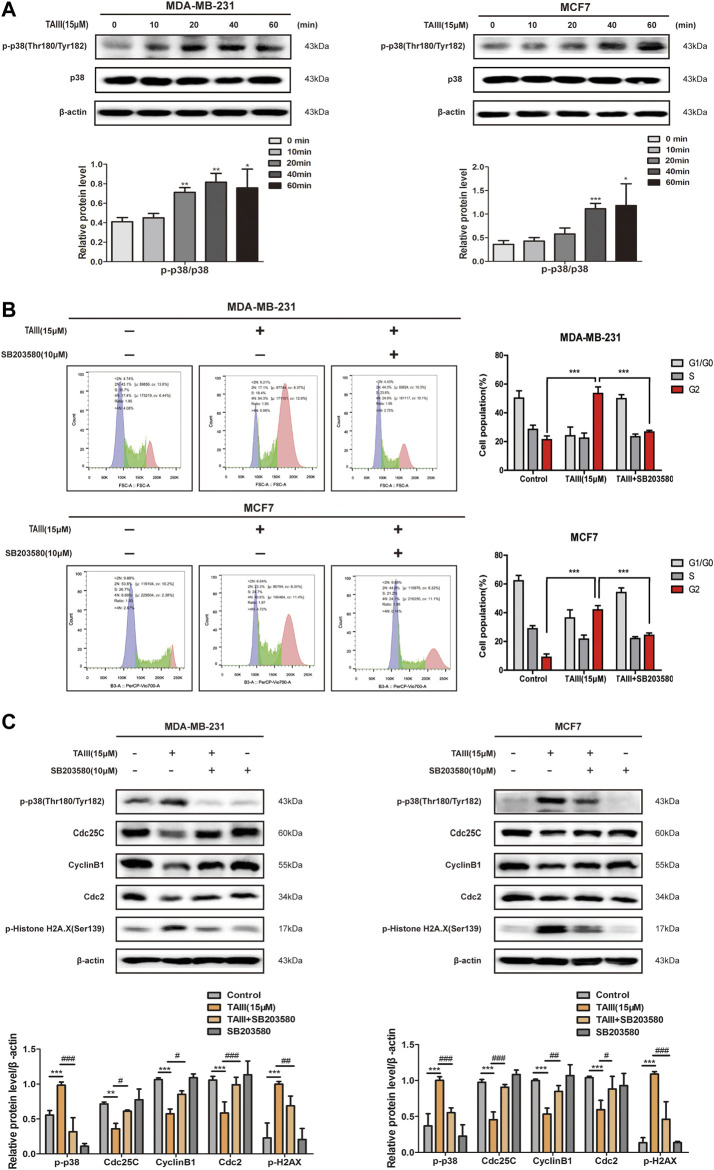
TAIII activates the p38 MAPK pathway and p38 specific inhibitor partial attenuates TAIII-induced G2 arrest in breast cancer cells. **(A)** MDA-MB-231 and MCF-7 cells were treated with 15 μM TAIII for 0, 10, 20, 40, and 60 min respectively and the expression of p-p38 were detected by western blot. **(B)** MDA-MB-231 and MCF-7 cells were pretreated with/without 10 μM SB203580 for 2 h before treated with 15 μM TAIII for 24 h and the cell cycle distribution (percent cells) was analyzed by MACSQuant Analyzer 10 after PI-staining, and data analyzed with FlowJo_V10 software. **(C)** MDA-MB-231 and MCF-7 cells were pretreated with/without 10 μM SB203580 for 2 h before treated with 15 μM TAIII for 24 h and the expression of p-p38, p-Histone H2A.X, Cdc25C, CyclinB1 and Cdc2 were detected by western blot. β-actin was used as the loading control. The data were expressed as the mean ± SD of the results from three separate experiments and the differences between groups was analyzed by *t*-test, two-way analysis of variance (ANOVA). *p < 0.05; **p < 0.01; ***p < 0.001.

It has been demonstrated that activation of p38 MAPK pathway induce cell cycle arrest at G2/M phase through increasing the expression of the inhibitory phosphorylation of Cdc25C at Ser216 (Bulavin et al., 2001; Garner et al., 2002; Pedraza-Alva et al., 2006). To determine whether the p38 MAPK pathway plays a role in TAIII-induced G2/M phase arrest in MDA-MB-231 and MCF7 cells, expressions of phosphorylation of p38 at Thr180/Tyr182 were examined. As shown in Figure 5A, a time-dependently increased of phosphorylation of p38 at Thr180/Tyr182 was observed in both MDA-MB-231 and MCF7 cells.

To further clarify the role of the p38 MAPK pathway in TAIII-induced G2/M phase arrest, MDA-MB-231 and MCF7 cells were pretreated with/without 10 μM SB203580 (a inhibitor of p-p38) for 2 h before TAIII treatment. Results showed that TAIII-induced G2/M phase arrest was partially reversed by SB203580 (Figure 5B). The percentage of MDA-MB-231 cells in G2/M phase was decreased from 53.43% in the TAIII group to 26.66% in the TAIII+SB203580 group. For MCF7 cells, the percentage of cells in G2/M phase was decreased from 41.94% in the TAIII group to 24.27% in the TAIII+SB203580 group. Meanwhile, TAIII-induced changes in the expressions of Cdc25C, CyclinB1, Cdc2 and γ-H2AX were partially restored by SB203580. It is noticed that the declined expression of γ-H2AX was observed after cells were pretreated with SB203580, which indicated that p38 MAPK pathway also played a role in DNA damage. Collectively, these data confirm that activation of p38 MAPK pathway is involved in TAIII-induced G2/M arrest in breast cancer cells.

### TAIII Inhibits the Growth of Breast Cancer Xenografts in Nude Mice

**FIGURE 6:**
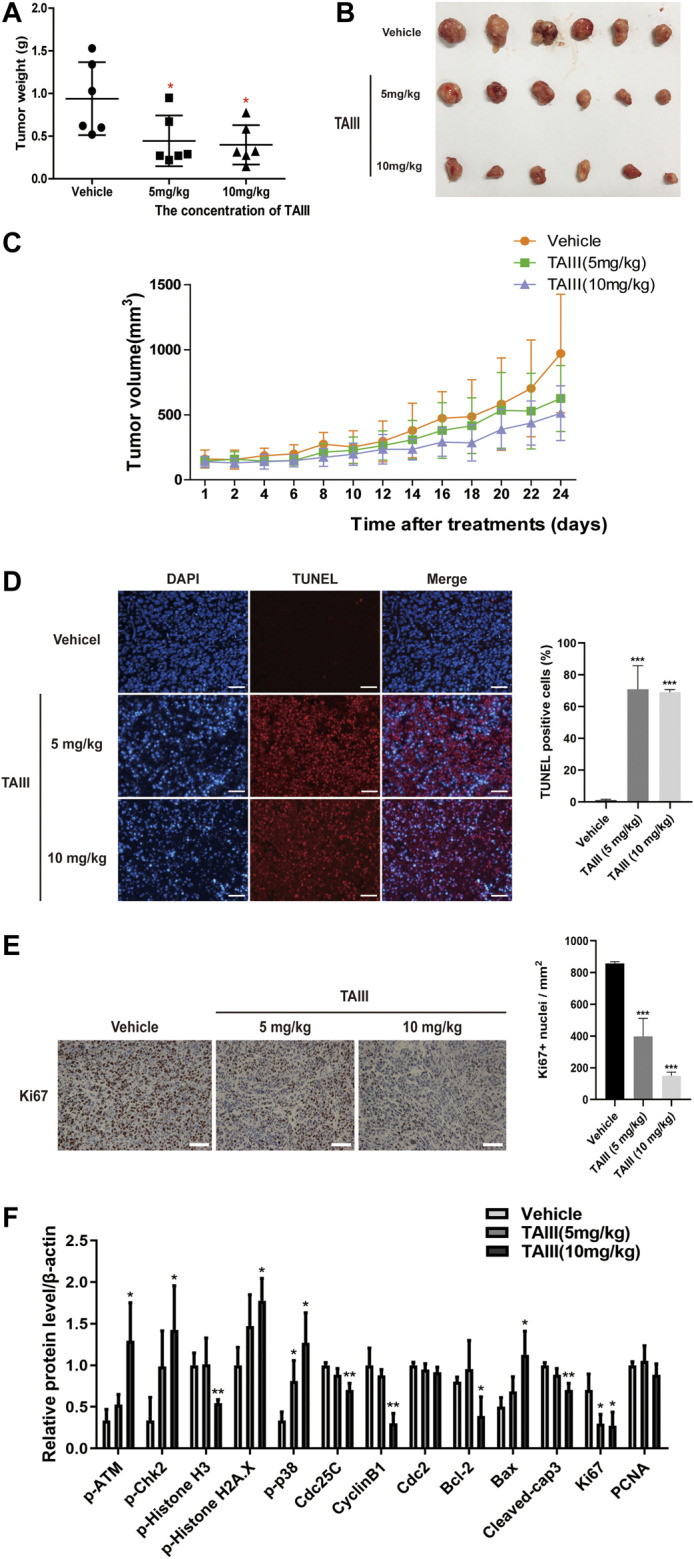
TAIII inhibits the growth of breast cancer xenografts in nude mice. **(A)** Tumor weight changes of mice in each group. **(B)** Photograph of tumors excised from mice at day 24. Day 0 is the day of first injection. **(C)** Tumor size changes of mice in each group during TAIII treatment. Data are presented as mean ± SD (*n* = 6). The *t*-test in MDA-MB-231 subcutaneous tumor model, *p < 0.05; **p < 0.01; ***p < 0.001. **(D)** TUNEL assay was performed to detect cell apoptosis in tumor tissue. For each panel, the images (left to right) show nuclei stained by DAPI (blue), DNA fragment stained by TUNEL (red), and overlays of the two images. Scale bar = 50 μm and magnification = ×200. **(E)** Immunohistochemical analysis of tumors with Ki-67 was performed to detect cell proliferation in tumor tissue. Scale bar = 50 μm and magnification = ×400. **(F)** Quantifications of the indicated proteins examined in western blotting assay. The data were expressed as the means ± SD of the results from three mice for each group and the differences between groups was analyzed by *t*-test, two-way analysis of variance (ANOVA). *p < 0.05; **p < 0.01; ***p < 0.001.

To further assess the potential anti-breast cancer effects of TAIII *in vivo*, MDA-MB-231 cells were injected subcutaneously into 5∼6-weeks female BALB/c-Nude mice to establish the subcutaneous xenograft models. The mice were grouped randomly into three groups and treated with physiological saline (containing 1% tween 80, 2% DMSO), 5 mg/kg and 10 mg/kg TAIII every other day by intraperitoneal injection. At the same time, the volume of tumor and the body weight of the mice were recorded. As shown in Figures 6A,B, the tumor weight of the TAIII treated group was significantly smaller with 0.445 g (TAIII 5 mg/kg) and 0.398 g (TAIII 10 mg/kg), compared with untreated group as 0.940 g, which was consistent with the tumor volume data (Figure 6C). Meanwhile, no significant reduction in body weight of mice was observed in TAIII treated groups compared with the vehicle group (Supplementary Figure S1A). The pathological alternations of major organs from the mice in all groups were observed by H&E staining. As shown in Supplementary Figure S1B, no obvious pathological features were observed in TAIII-treated mice. The integrated results indicate that TAIII exerts a potent inhibitory effect on tumor growth *in vivo*.

Next, we detected the mechanism underlying the anti-breast cancer effects of TAIII *in vivo*. TUNEL assay was emploied to assess the apoptosis in paraffin-embedded tumor sections. As shown in Figure 6D, tumors from TAIII-treated mice (5 mg/kg or 10 mg/kg) showed a larger proportion of cells with TUNEL-positive nuclei than those from vehicle-treated mice (*p* < 0.001), which demonstrated that TAIII could induce apoptosis in xenograft tumor cells. Meanwhile, Ki67 staining showed that TAIII suppressed cell proliferation *in vivo* (Figure 6E).

For the changes of related-protein expression corresponding to *in vitro* data, the DNA damage related proteins and p38 pathway were mainly examined. As shown in Figure 6F and Supplementary Figure S2, increased expressions of phosphorylation of ATM at Ser1981, phosphorylation of Chk2 at Thr68 and γ-H2AX were observed in TAIII treatment groups and a decreased expression of phosphorylation of Histone H3 at Ser10 was observed. Meanwhile, p38 MAPK pathway was activated after TAIII treatment *in vivo*. Compared to the vehicle group, the expressions of Cdc25C and CyclinB1 in the tumor tissues were significantly decreased in TAIII-treated groups. These data indicate that TAIII induces G2/M arrest *in vivo*.

## Discussion

TAIII is a steroidal saponin compound isolated from the ethanol extract of *Anemarrhena asphodeloide*, and its remarkable antitumor activities have attracted the attention of many scholars, which are associated with the induction of autophagy, cell cycle arrest, apoptosis and suppression of migration and invasion (Takeda et al., 2001; Sy et al., 2008; King et al., 2009; Kang et al., 2011; Tsai et al., 2013; Wang et al., 2013; Huang et al., 2015; Jung et al., 2016; Kim et al., 2016; Nho et al., 2016; Wang et al., 2017; Gergely et al., 2018; Marelia et al., 2018). Here, we find that TAIII triggered DNA damage through activating the ATM/Chk2 and p38 MAPK signaling, leading to G2/M arrest and apoptosis in breast cancer.

The cell cycle distribution of cells was analyzed with PI staining by flow cytometry after treatment with different concentration of TAIII. Results showed that TAIII induced G2/M arrest in breast cancer cells (Figure 1A). The complex of CyclinB1 and Cdc2, or MPF is the engine molecules of cells entering M phase from G2 phase. CylinB1 is periodically expressed and degraded. It is synthesized in S phase, peaks in the G2 phase and degraded in the late M phase. Cdc2 is continuously synthesized in the cell cycle with stable content and its kinase activity is the key to driving the progression from G2 to mitosis. CyclinB1 helps Cdc2 to localize in the nucleus to convey transcription information (Okumura et al., 1996). However, phosphorylation of Cdc2 at Tyr15 prevents Cdc2 from binding to CyclinB1, that is, phosphorylation of Cdc2 at Tyr15 hinders the formation of MPF. An increased expression of inhibitory phosphorylation of Cdc2 at Tyr15 and decreased expression of Cdc2 and CyclinB1 were observed by TAIII (Figures 1B,C, 3), indicating that TAIII induces the G2/M phase arrest by inhibiting the formation of MPF in breast cancer cells.

The activity of Cdc2 reflects the activity of the complex, which is regulated by various pathways. Once the cell decides to divide, Cdc25C activates Cdc2 by dephosphorylating the Cdc2 at Tyr15 and Thr14 (Hoffmann and Karsenti, 1994; Schafer, 1998). In other words, the inhibition of Cdc25C activation fails to dephosphorylate Cdc2, which probably leads to cell cycle arrest at G2/M phase. Our results showed that the activity of Cdc25C was inhibited significantly by TAIII in a concentration- and time-dependent manner (Figures 1B,C), accompanied with the inhibition of Cdc2, then the formation of MPF was inhibited and finally induced G2/M phase arrest in breast cancer cells, suggesting TAIII affects coherent pathways that regulate Cdc25C kinase activity. Previous studies have shown that the activity of Cdc25C is related to the DDR pathway and p38 pathway (Maity et al., 1994; Bulavin et al., 2001; Garner et al., 2002; Pedraza-Alva et al., 2006).

DDR pathway coordinates the detection and repair of DNA damage to ensure the maintenance of genomic stability and cell viability (Jackson and Bartek, 2009; Ciccia and Elledge, 2010). Once activated, the DDR pathway inhibits the expression of cell cycle-related regulatory proteins and causes cell cycle arrest to detect and repair DNA damage. Several literatures report that ATM is activated by phosphorylation at Ser1981, accompanied by activation of Chk2 (phosphorylation at Thr68) (Matsuoka et al., 1998; Falck et al., 2001). The activation of Chk2 phosphorylates Cdc25C at Ser216, which inhibits the activity of Cdc25C and the downstream protein Cdc2 and activates the G2/M phase checkpoints (Matsuoka et al., 1998; Shiloh, 2001; 2003). γ-H2AX triggers a cascade reaction and recruits repair proteins at the DNA damage site to repair DNA damage (Burma et al., 2001), which is a “crosstalk” in DNA damage repair, a sign of DNA damage, and an important mechanism of DDR (Van Attikum and Gasser, 2009). Our results showed that TAIII activated the DDR pathway (ATM/Chk2/Cdc25C) and γ-H2AX quickly (Figure 3), indicating that TAIII induces DNA damage in breast cancer cells. If DDR cannot repair DNA damage accurately, it probably induces cell senescence or apoptosis (Shiloh, 2003). Based on the experimental results, we speculate that DDR fails to repair TAIII-induced DNA damage, so the breast cancer cells were arrested the G2/M phase permanently and apoptosis occurred eventually. After blocking the DDR pathway, it was found that the percentage of cells in the G2/M phase was declined (Figure 4A), which confirmed that TAIII-induced breast cancer cells arrest at G2/M phase arrest was related to the activation of the DDR pathway.

ATM and ATR are activated by different types of DNA damage and function in different ways. Their activation depends on the type of genotoxic stress, and the activation of ATM is specific to DNA double-strand breaks (DSBs) (Helt et al., 2005). ATM is a major kinase involved in the phosphorylation of γ-H2A.X at Ser139 (Burma et al., 2001). Phosphorylated γ-H2A.X stabilizes double-stranded DNA breaks and provides binding sites for DNA-related repair proteins. It is the central component of the DSB reaction and is extremely important for the repair of DSBs. Therefore, γ-H2A.X is also considered as a hallmark of DSBs (Rogakou et al., 1998; Downs et al., 2000; Pilch et al., 2003). TAIII-activated the ATM/Chk2 pathway and the γH2AX are specifically response to the repair of DSBs (Figures 4A, 6F), so we speculate that the type of DNA damage induced by TAIII in breast cancer cells is DSBs.

In response to DSBs, p38 MAPK is activated and leads to the establishment of a G2/M cell cycle checkpoint (Bulavin et al., 2002; Mikhailov et al., 2004). Our results showed that TAIII activated p38 MAPK pathway both in MDA-MB-231 and MCF7 cells, and the TAIII-induced G2/M phase arrest was partially attenuated by SB203580 (Figure 5). Meanwhile, the activation of p38 MAPK pathway was also observed in TAIII-treated tumor tissues (Figure 6F). These results demonstrate that TAIII-induced DSBs causes activation of p38 MAPK, and then lead to G2/M cell cycle arrest. It has been reported that p38 MAPK activation in response to DNA DSBs is mediated by ATM-dependent pathway and other pathways (Mikhailov et al., 2004; Raman et al., 2007; Reinhardt et al., 2007). For ATM-dependent pathway, ATM does not appear to be able to directly phosphorylate p38 MAPK, and activation of p38 MAPK appears to be mediated by the Thousand and one (Tao) kinases, which are directly activated by ATM. Next, p38 MAPK can induce a G2/M checkpoint through the phosphorylation and inhibition of the phosphatase Cdc25B (Bulavin et al., 2001) and Cdc25C (Hirose et al., 2003; Wang et al., 2017). Our results demonstrate that TAIII activates both ATM and p38 MAPK pathways, then inhibits Cdc25C level, inducing G2/M phase arrest in breast cancer cells.

In addition, CKI is an important component of negative regulation of the cell cycle, and its family member p21 is mainly responsible for the regulation of G2/M phase. Our data showed that TAIII increased expression of p21 in MDA-MB-231 cells and decreased expression of p21 in MCF-7 (Figure 1B). While the tumor suppressor gene p53, which directly regulates p21, plays an important role in DNA damage. It is reported that G1 phase arrest in cells is p53-dependent (Little et al., 1995), and the effect of p53 on G2/M phase arrest is controversial. Studies have shown that G2/M phase arrest can be induced in p53-defective cells (Yao et al., 1996), in other words, p53 is not necessary for inducing G2/M phase arrest in cells. It is reported that induction of G2/M phase arrest does not all depend on p21 (Casagrande and Darbon, 2000). Thus, we suspect that the expression of p21, which is the target gene of p53, is affected by TAIII differently in MDA-MB-231 (mutant p53) and MCF7 (wide type p53) cells due to the different status of p53 in the two cell lines. This might also account for the difference in the degree of G2 phase arrest induced by TAIII. Plus, compared to the transient inhibition of CyclinB1/Cdk1 complex by Chk1/Chk2, p21-mediated premature activation of APC/C-Cdh1 leads to the degradation of CyclinB1 and other mitotic regulators. That is, the inhibition of CyclinB1/Cdk1 complex by p21 is complete and irreversible (Baus et al., 2003). FCM and WB showed that the TAIII-induced G2/M arrest was partially reversed after blocking the ATM/Chk2 and p38 pathways, which also confirms this point. It is also reported that p21 is not critical for the regulation of G2/M arrest (Abraham, 2001).

Cell senescence and apoptosis are two important mechanisms for inhibiting genetically abnormal cell proliferation. Cell senescence is induced by DNA damage, oncogenic signal transduction and telomere shortening (Kuilman et al., 2010; Salama et al., 2014). And DDR and oncogene-induced senescence (OIS) are often found in precancerous lesions and are considered to constitute obstacles to tumorigenesis. Recently, a large number of studies have shown that G2/M phase arrest can cause cell senescence (Verdun et al., 2005; Leontieva et al., 2012; Mao et al., 2012; Jullien et al., 2013; Ye et al., 2013), suggesting that G2/M rather than G1/S checkpoints may be the reason for replicative senescence (Chassot et al., 2008; Spencer et al., 2013). Gergely J et al. found that TAIII significantly inhibits the proliferation, migration and invasion of breast cancer cells and induces the senescence of breast cancer cells by inhibiting he activity of polycomb repressive complex 1 (the core protein of polycomb complex) histone post-translational modification and reducing the expression of its core member BMI1 in breast cancer cells (Gergely et al., 2018). Combining the connection between G2/M phase arrest and cell senescence, the relationship between DDR pathway and cell senescence, the research of Gergely J and the comprehensive mechanism of TAIII on breast cancer cells in this article, we considered that TAIII exerts the anti-breast cancer effects not only by inducing G2/M phase arrest to inhibiting the proliferation and senescence in breast cancer cells, but also by strengthening the DDR pathway to prevent the tumor immune escape.

In summary, we provide compelling evidence demonstrating that TAIII induces cell cycle arrest and apoptosis through ATM/Chk2 and p38 MAPK signaling pathways, associating with induction of DNA damage, which offer a promising anti-tumor agent against breast cancer. Certainly, with a low toxicity and high selectivity in cancer cells, TAIII can be applied in combination therapy. However, the mechanisms by which TAIII induces DNA damage and the crosstalk with other reported pathways regulated by TAIII are not defined here, which will be studied in our future work.

## Data Availability

The raw data supporting the conclusions of this article will be made available by the authors, without undue reservation.
