# Supplementary figures and images for: Timosaponin AIII Induces G2/M Arrest and Apoptosis in Breast Cancer by Activating the ATM/Chk2 and p38 MAPK Signaling Pathways
Source: Front Pharmacol. 2021 Jan 15;11:601468. doi: 10.3389/fphar.2020.601468 (PMC7898553; doi:10.3389/fphar.2020.601468)

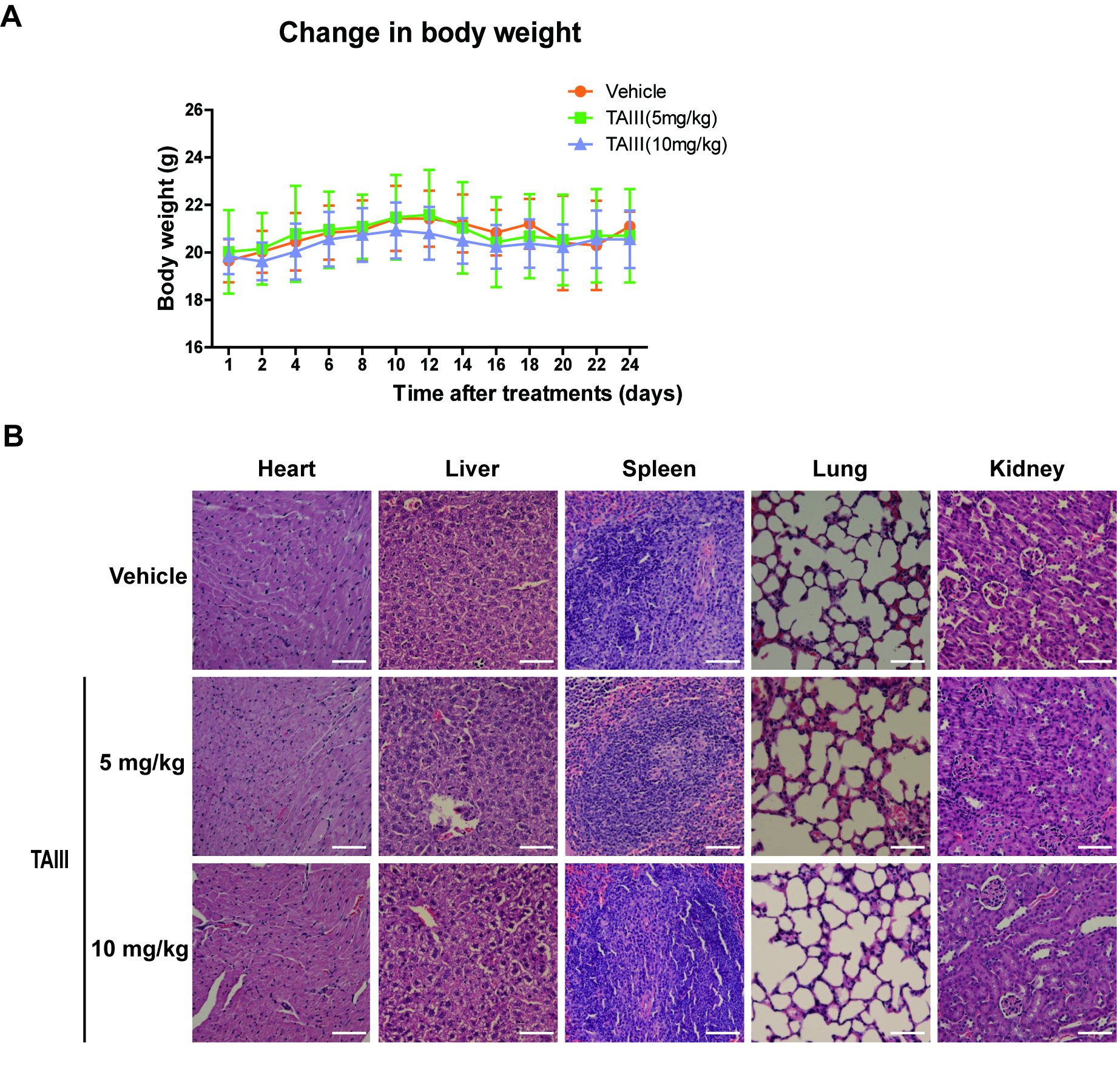

Supplement: Supplementary file 1 [file image1.tif]

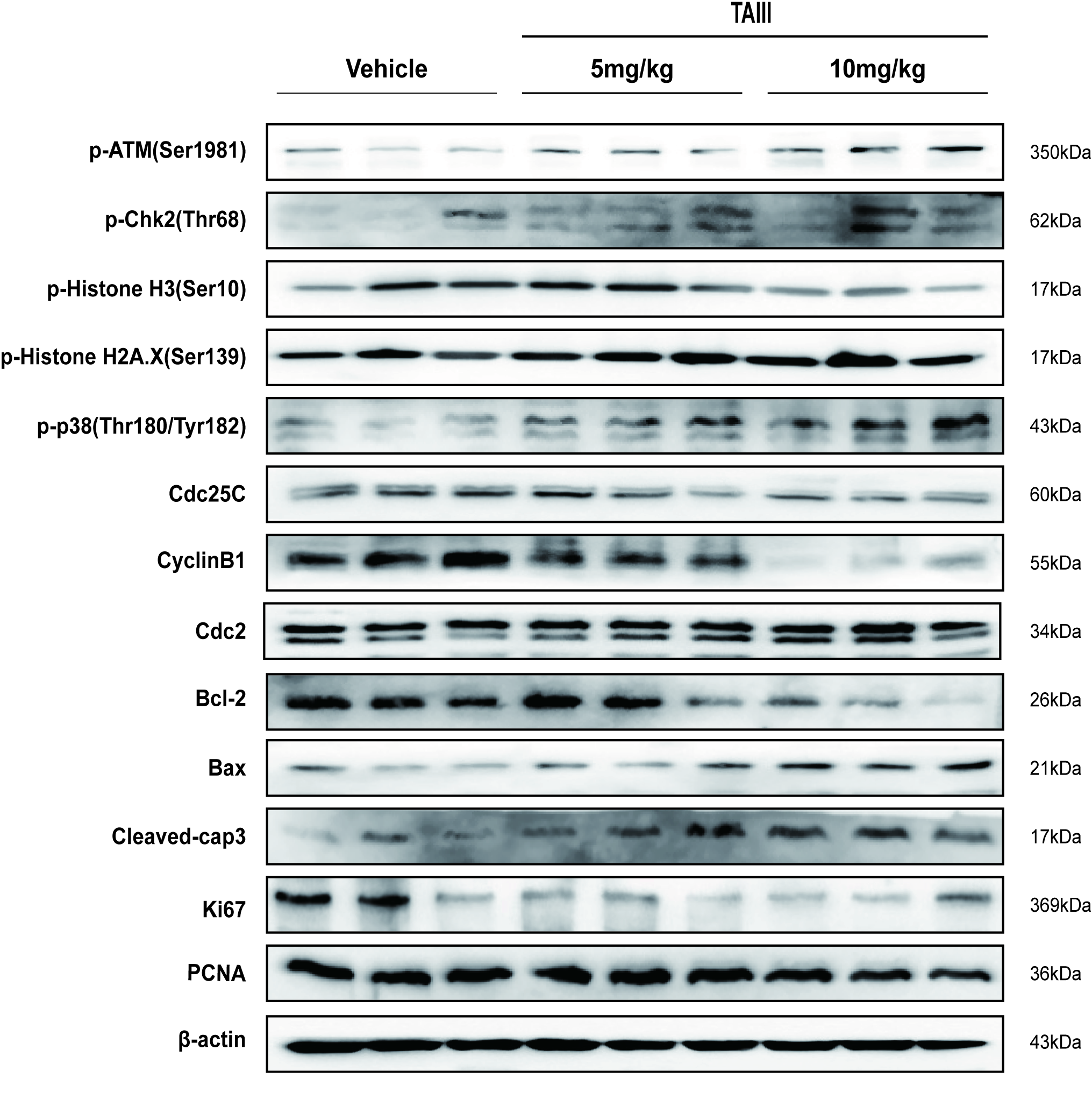

Supplement: Supplementary file 2 [file image2.tif]
